# Autophagy and proliferation are dysregulated in Charcot-Marie-Tooth disease type 2A cells harboring MFN2 (mitofusin 2) mutation

**DOI:** 10.1080/27694127.2022.2132447

**Published:** 2022-10-11

**Authors:** Paola Zanfardino, Vittoria Petruzzella

**Affiliations:** Department of Translational Biomedicine and Neurosciences, University of Bari Aldo Moro, Bari, Italy

**Keywords:** AKT, autophagy, cell proliferation, Charcot-Marie-Tooth type 2A, mitochondria, mitofusin 2, mitophagy, transcriptomics

## Abstract

MFN2 (mitofusin 2) is a mitochondrial outer membrane protein that serves primarily as a mitochondrial fusion protein, which is its best known role but has additional functions in regulating cell biological processes. Multiple functions include participation in mitochondrial fusion, tethering of mitochondrial-endoplasmic reticulum membranes, movement of mitochondria along axons, and control of the quality of mitochondria, which is important for the maintenance of cellular homeostasis. Mitochondrial quality control is a process that includes the exchange of mitochondrial components through mitochondrial fusion and fission, and the removal of dysfunctional mitochondria through autophagy/mitophagy. Macroautophagy/autophagy, as major intracellular machinery for degrading aggregated proteins and damaged organelles, is involved in the occurrence of pathological changes in diabetes, obesity, neurodegenerative diseases and cancer. Intriguingly, MFN2 has been referred to as a tumor suppressor gene in some forms of cancer. Several studies of the effects of MFN2 mutations have not been conclusive on molecular mechanisms causing cellular alterations. We tackled some of these issues in fibroblasts derived from a Charcot-Marie-Tooth disease type 2A (CMT2A) patient with a mutation in the GTPase domain of MFN2. So, in this *punctum*, we discuss the mechanism whereby mitochondrial MFN2 protein mutation affects autophagy and cell proliferation rate.

## Effect of dominant-negative MFN2 monoallelic mutation on mitochondrial functionality

CMT2A disease (OMIM 609260), is an autosomal dominant inherited sensorimotor neuropathy affecting peripheral nerve axons, that has causative mutations in the *MFN2* gene. Discordant data on mitochondrial alterations in fibroblasts derived from patients with MFN2 mutations and animal models have been reported. Moreover, most of the studies on the role of MFN2 in autophagy have been performed in tumor cells where autophagy has a dual significance in response to diverse nutrient conditions, possibly acting as either a tumor-suppressor or -promoting pathway. We identified a monoallelic *MFN2* 650G>T/MFN2^C217F^ mutation in the GTPase domain in a CMT2A (CMT2A^MFN2^) patient [1]. Although we did not perform a direct measurement of mutated and wild-type (WT) forms to assess their relative abundance, we found that both the MFN2 protein and mRNA levels are unchanged. Deep-sequencing analysis of the *MFN2* mRNA in the CMT2A^MFN2^ fibroblasts showed a similar content of mutated and WT *MFN2* mRNA which led us to hypothesize that also as proteins, the mutated and wild-type forms may coexist. Considering that MFN2 functions as a dimer either with itself or with MFN1, an MFN2 homolog, we can hypothesize that the monoallelic mutation acts as a dominant-negative trait on mitochondrial function. Indeed, we observed mitochondrial network fragmentation, organelle depolarization, and impaired respiration due to a significant reduction of respiratory complexes’ activities in the patient’s cells, all conditions that would be consistent with the dominant-negative effect of the mutant allele. CMT2A^MFN2^ fibroblasts presented an increase in the so-called intermediate-fragmented mitochondria and have an inefficient capacity to recover the morphology upon removal of a stressful insult. Hence, we asked whether the presence of damaged mitochondria in CMT2A^MFN2^ cells would promote their clearance through the stimulation of autophagy and mitophagy.

We examined the autophagy pathway in CMT2A fibroblasts evaluating the expression of two autophagic markers, SQSTM1/p62 (sequestosome 1), which binds to ubiquitinated substrates and targets them to phagophores, and MAP1LC3/LC3 (microtubule associated protein 1 light chain 3), a protein that plays a role in autophagosome maturation. We find decreased LC3B-II protein levels, which suggests a reduction of autophagosome formation. As LC3 is required for autophagosome synthesis and maturation, a decrease in its levels may suggest the derailment of autophagy initiation. Conversely, because the SQSTM1/p62 protein levels are increased, we hypothesized an abnormal clearance within the cells due to a low autophagic response that poses detrimental effects in mutated fibroblasts. This hypothesis was supported by the discovery of the downregulation of *RAB37* mRNA in the CMT2A^MFN2^ fibroblasts, which regulates LC3B lipidation. It would be worth investigating if our finding of the reduced conversion of LC3B-I to LC3B-II is indeed related to the reduced expression of RAB37.

Next, we investigated whether, despite the low autophagic response, the dysfunctional mitochondria are properly tagged and recruited for digestion and elimination by mitophagy. We found that despite an absolute lower number of autophagosomes in mutated CMT2A^MFN2^ compared to the normal control fibroblasts, a larger number of them are engaged in engulfing mitochondria in the former, indicating that mitophagy is increased in CMT2A^MFN2^ fibroblasts. To our knowledge, this is the first evidence reporting that despite defective initial steps of autophagy, proper recruitment of the functionally impaired mitochondria for elimination by mitophagy takes place in CMT2A^MFN2^ fibroblasts. Because we have not established whether all damaged mitochondria present in cells can be sufficiently eliminated by the low number of autophagosomes in CMT2A^MFN2^ cells, we cannot rule out that damaged mitochondria may induce other mechanisms to prevent their accumulation within the cell.

## AKT: a new potential actor in CMT2A2 pathophysiology

We observed that CMT2A^MFN2^ fibroblasts show a consistent acceleration of cell division. The transcriptomic analysis helped us to shed light on the molecular pathways responsible for the dysfunctions found in mutated CMT2A^MFN2^ fibroblasts. Most of the differentially expressed genes are enriched in cell population proliferation, extracellular matrix organization, and the phosphoinositide 3-kinase (PI3K)-AKT signaling pathway. Interestingly, we find upregulation of IRS2 (insulin receptor substrate 2) and IGF1 (insulin like growth factor 1), two known upstream regulators of the PI3K-AKT pathway. Transcripts encoding key factors involved in positive regulation of PI3K signalling, which acts upstream of MTOR (mechanistic target of rapamycin kinase), are also significantly altered. Because the serine/threonine-protein kinase AKT is an important signal transducer of the PI3K pathway in all cells and tissues, we studied AKT activation and consistently find MTORC2-AKT signaling activation. Consequently, the opposite effects on autophagy and proliferation, the downregulation of autophagy initiation and the increase of the cell proliferation rate of CMT2A^MFN2^ fibroblasts, agree with the known anabolic effects of activation of the PI3K-AKT-MTOR pathway. To highlight the AKT involvement, we used a potent and selective allosteric AKT inhibitor (miransertib). When we evaluated the effect of the pharmacological treatment on autophagosome formation and cell proliferation we found that the decreased autophagy and increased cell proliferation can be attenuated by inhibiting AKT. Taken together, all these data show the dependence of both autophagy and cell proliferation on the AKT pathway and that drug treatment restores both autophagy and cell proliferation rate in CMT2A^MFN2^ cells. Because AKT activation is regulated by MFN2 itself through MTORC2 activation, we can infer that MFN2 mutation drives these aspects. Therefore, this work unveils a novel potential role of AKT in the pathophysiology of CMT2A2.

**Figure 1.**
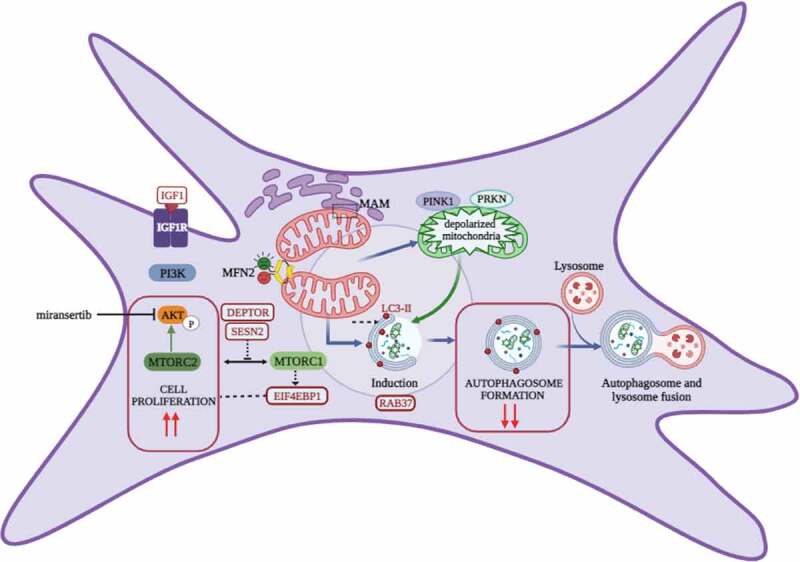
Decreased autophagy and increased proliferation are features of CMT2A^MFN2^ fibroblasts. MFN2 presents multiple functions that include participation in mitochondrial fusion, tethering of mitochondrial-endoplasmic reticulum membranes (MAM: mitochondrial-associated membranes), and control of the quality of mitochondria. The mutant MFN2 (in green) can dimerize with the wild-type MFN2 (in red) in CMT2A fibroblasts and can induce mitochondria depolarization and damage. Depolarized mitochondria (in green) are recruited for elimination by mitophagy although CMT2A^MFN2^ fibroblasts present a downregulation of autophagy initiation. CMT2A fibroblasts also show increased cell proliferation. The AKT-MTOR pathway regulates both autophagy and proliferation. AKT activation requires several kinases: MTORC2 phosphorylates AKT at Ser473 leading to cellular proliferation. AKT can activate cellular proliferation either directly or via MTORC1 that acts on EIF4EBP1 and inhibits autophagy either by an MTORC1-dependent or -independent pathway. The decreased autophagy and increased cell proliferation can be attenuated by inhibiting AKT through drug treatment (miransertib). Red circles highlight genes found differentially expressed in transcriptomic experiments in CMT2A^MFN2^ fibroblasts: IGF1, SESN2, DEPTOR (negative regulators of MTOR) and EIF4EBP1. Interestingly, IGF1 is an activator of the PI3K-AKT-MTOR pathway.

In conclusion, our evidence indicates that MFN2 mutation can positively drive cell proliferation in CMT2A^MFN2^ fibroblasts and that increased proliferation can be useful for CMT2A^MFN2^ cells to clear them of damaged mitochondria as a further path to autophagy (Figure 1). In the future, it would be worth investigating if the mutated MFN2 interferes with autophagy and the cell division rate of neuronal committed cells generated from CMT2A patients and/or with the differentiation of neural stem cells, which are more closely related to the specific cell types involved in the disease.

## References

[1] Zanfardino P, Longo G, Amati A, et al. Mitofusin 2 mutation drives cell proliferation in Charcot–Marie-Tooth 2A fibroblasts. Human Molecular Genetics 2022;ddac201. Online ahead of print. 10.1093/hmg/ddac201 35994048

